# Calcium-signaling proteins mediate the plant transcriptomic response during a well-established *Xanthomonas campestris* pv. *campestris* infection

**DOI:** 10.1038/s41438-019-0186-7

**Published:** 2019-09-11

**Authors:** Maria Tortosa, Maria E. Cartea, Pablo Velasco, Pilar Soengas, Victor M. Rodriguez

**Affiliations:** Group of Genetics, Breeding and Biochemistry of Brassicas, Misión Biológica de Galicia, Spanish Council for Scientific Research (CSIC), PO Box 28 E-36080 Pontevedra, Spain

**Keywords:** Biotic, Plant signalling

## Abstract

The plant immune system is divided into two branches; one branch is based on the recognition of pathogen-associated molecular patterns (PAMP-triggered immunity), and the other relies on pathogenic effector detection (effector-triggered immunity). Despite each branch being involved in different complex mechanisms, both lead to transcription reprogramming and, thus, changes in plant metabolism. To study the defense mechanisms involved in the *Brassica oleracea*–*Xanthomonas campestris* pv. *campestris* (Xcc) interaction, we analyzed the plant transcriptome dynamics at 3 and 12 days postinoculation (dpi) by using massive analysis of 3′-cDNA ends. We identified more induced than repressed transcripts at both 3 and 12 dpi, although the response was greater at 12 dpi. Changes in the expression of genes related to the early infection stages were only detected at 12 dpi, suggesting that the timing of triggered defenses is crucial to plant survival. qPCR analyses in susceptible and resistant plants allowed us to highlight the potential role of two calcium-signaling proteins, *CBP60g* and *SARD1*, in the resistance against Xcc. This role was subsequently confirmed using Arabidopsis knockout mutants.

## Introduction

Plant leaves are relatively isolated from the environment by physical barriers (i.e., the cuticle) that prevent desiccation and the penetration of phytopathogens. However, leaf metabolism requires gas and water interchange with the environment through pores that interrupt this barrier surface. These pores (stomata or hydathodes on the leaf margin), together with wounds in leaf tissues, are the entry points of pathogenic bacteria to the intercellular space where they can proliferate.

Unlike metazoans, plants lack mobile defender cells and an adaptive innate immune system, so plant defense relies on the capacity of individual cells to sense and respond to pathogens by reprogramming cell metabolism to induce the expression of defense genes^1^. Jones and Dangl^2^ model the plant cell immune system in a zigzag response organized at two different levels. The early basal response or PTI (Pathogen-associated molecular pattern (PAMP)-triggered immunity) is triggered upon the recognition of PAMPs on the cell surface and can prevent pathogen colonization of the cell^3^. However, compatible pathogens can overcome this first barrier of the immune system and trigger a second level of defense called ETI (effector-triggered immunity), mediated by NB-LRR proteins, which occurs mainly intracellularly^2,4^. The burst of these mechanisms of defense has also been associated with the activation of HR (hypersensitive response) and SAR (systemic acquired resistance)^5^. Several studies have revealed a high percentage of overlapping networks between the PTI and ETI phases^6,7^ to the extent that most authors consider the PTI a weak variant of ETI^8^. However, Pombo et al.^9^ reported that as little as 14% of the transcriptomic changes occur in response to the attack of *Pseudomonas syringae* on tomato plants are common between PTI and ETI responses. This apparent contradiction could be explained by the use of different plant-pathogen systems. Tsuda and Katagari^3^ reviewed the plant mechanisms in the response to different bacterial PAMPs and effectors, concluding that different PAMPs trigger the PTI response through common signaling pathways, whereas the cellular response to different pathogen effectors diverges among microbial types.

The immune response burst is driven by extensive transcriptional reorganization. Early response to pathogen recognition involves an increase in cytosolic secondary messengers to amplify the immune response through the activation of transcription factors. Increased intercellular Ca^+2^ levels along with ROS production play a pivotal role in this response. Many Ca^+2^ sensors have been described in land plants that perceive changes in cytosolic Ca^+2^ levels and transduce it into a downstream signaling response^10^. Intracellular Ca^+2^ sensors are represented by three families, i.e., calmodulin (CaM) and calmodulin-like proteins (CMLs), Ca^+2^-dependent protein kinases (CPKs) and Ca^+2^ and calmodulin dependent protein kinases (CCaMK)^11^. These three families can be grouped into two types: sensor relays (CaM and CMLs) and sensor responders (CPKs and CCaMK). Sensor relays function through bimolecular interactions, whereas sensor responders function through intramolecular interactions^12^. However, the mechanism by which plant sensors modulate the plant response to different stimuli remains elusive. The specificity of the Ca^+2^-mediated response can be reached at several levels (for review see ref. ^13^), which includes the spatial and temporal transcriptional regulation of genes involved in the Ca^+2^ downstream signaling network.

With the aim of deciphering the molecular mechanisms involved in the response to bacterial pathogenesis, we investigated the transcriptome dynamics of *Brassica oleracea* in response to *Xanthomonas campestris* pv. *campestris* (Xcc) race 1 infection at 3 and 12 days after inoculation.

## Results

### Global changes in the *B. oleracea* transcriptome after Xcc infection

**Fig. 1:**
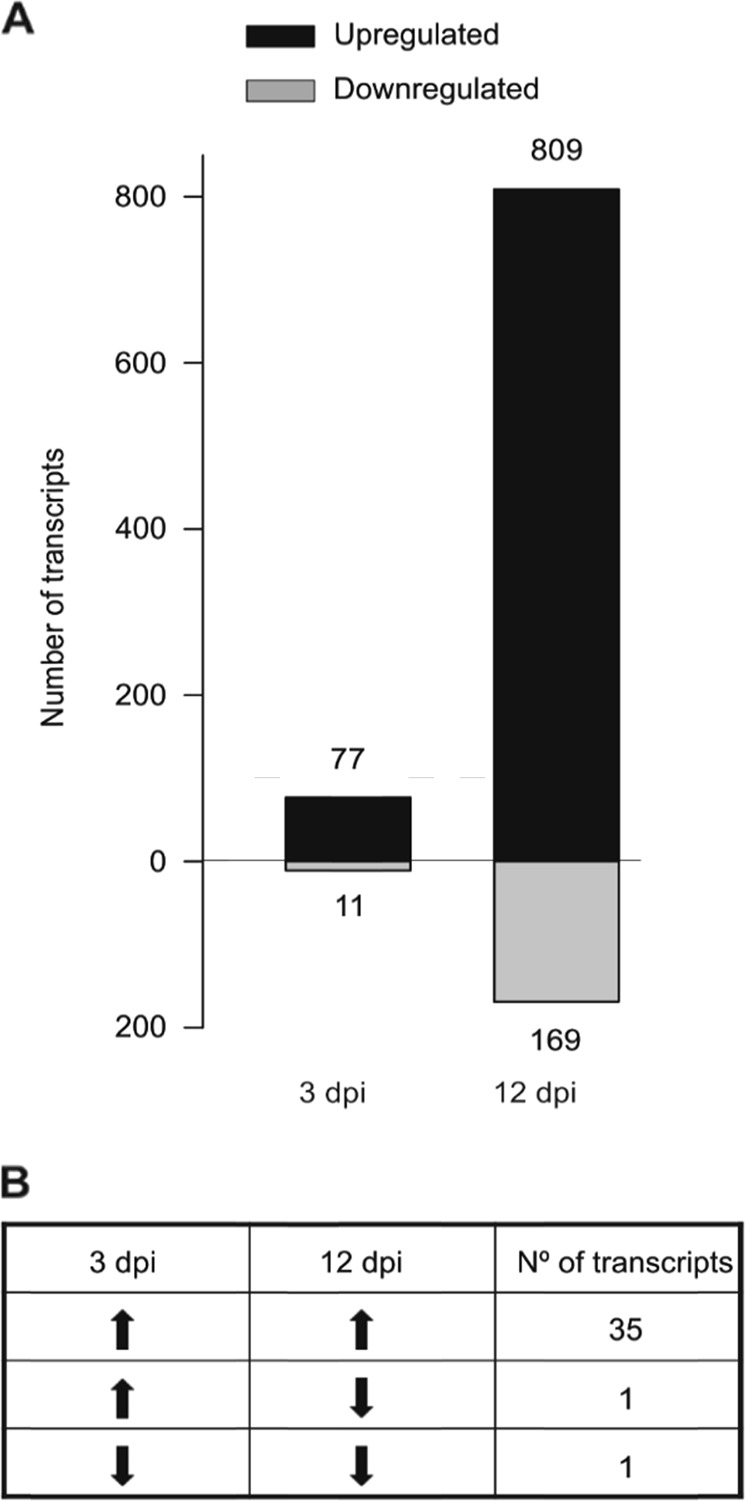
A higher number of genes were induced in *Brassica oleracea* at 12 dpi than at 3 dpi upon *Xanthomonas campestris* pv. *campestris* infection. **a** Total number of transcripts up- and downregulated at 3 and 12 dpi (Inoculated vs. Control). **b** Number of common transcripts identified at 3 and 12 dpi (Inoculated vs. Control)

To identify the genes that were upregulated and downregulated after Xcc infection, we performed a MACE analysis. We selected two different time points (3 and 12 dpi) based on previous observations. At 3 dpi, small necrotic lesions were observed at the inoculation points, whereas at 12 dpi, lesions reached the leaf edge and the midrib (Fig. S1). Statistical analysis was carried out within each time point to compare the transcriptomic expression of inoculated vs. control *B. oleracea* plants. An average of 217,132 tag sequences was read from each sample, and 77,637 of them were unambiguously annotated to the databases. We identified a higher transcriptomic response at 12 than at 3 dpi, albeit in both harvest times, the number of induced transcripts was higher than the number of repressed transcripts (Fig. 1a). Among the differentially expressed transcripts between the conditions, 37 were coregulated at both times (Fig. 1b). We identified three major functional groups among these genes. The major group corresponded to transcripts involved in phytohormone metabolism, which included important genes in the synthesis (lipoxygenases (LOX) or allele oxide synthase (AOS)) and perception (JAZ-proteins) of jasmonic acid (JA) and two methyl-transferases involved in the synthesis of methyl salicylate, an active form of salicylic acid (SA). It is commonly accepted that phytohormones are rapidly and transiently activated after a pathogenic attack; however, we found marked levels of these transcripts at 12 dpi. The other two groups encompassed typical genes involved in plant defense mechanisms (pathogenesis-related (PR) proteins and secondary metabolism activation, such as GSLs and flavonoids).

### Altered pathways during early bacterial infection

**Fig. 2:**
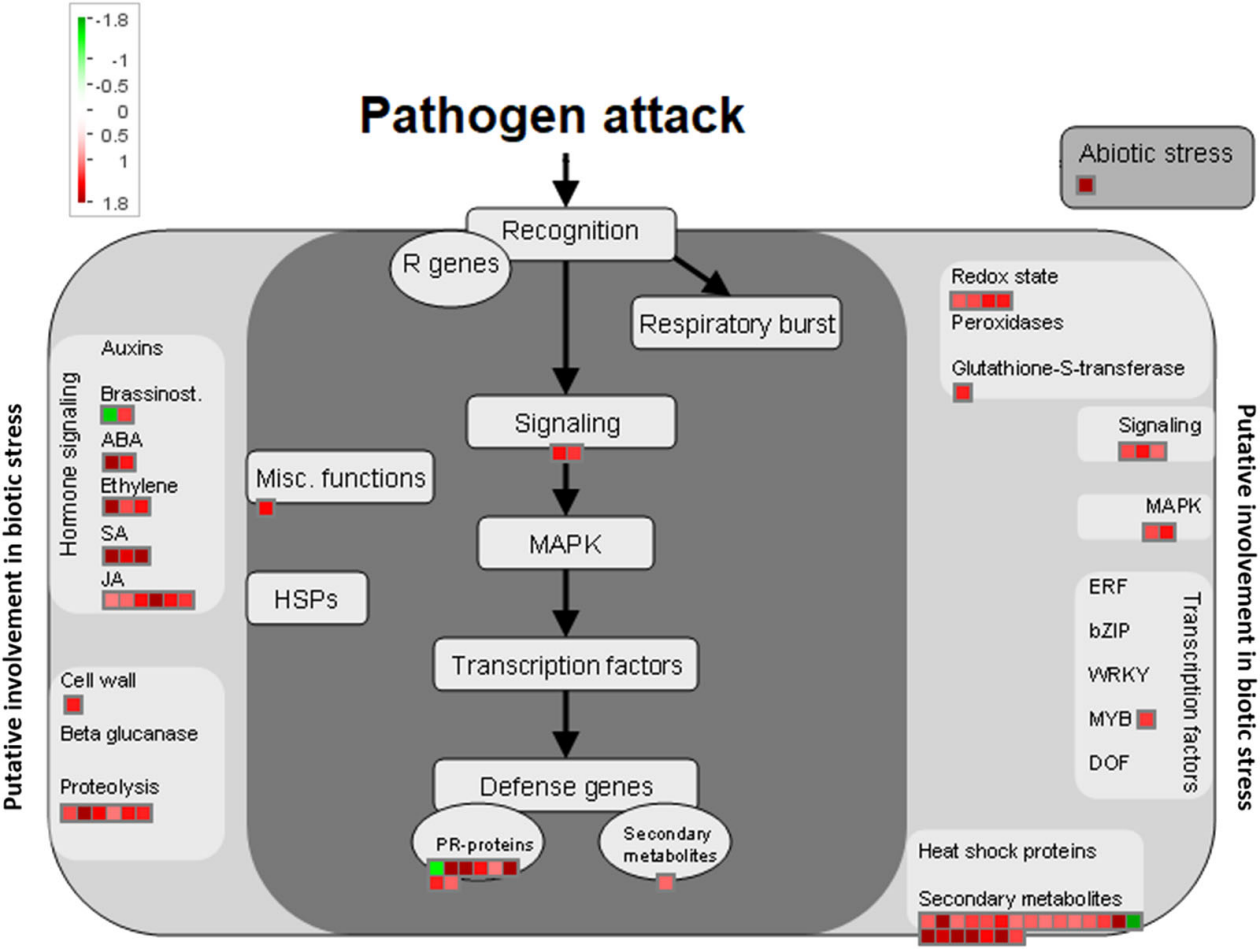
Distribution of the differentially expressed transcripts at 3 dpi and involved in biotic stress processes by using Mapman software. Green square: downregulated genes; red square: upregulated genes

The transcriptomic response during the first days after bacterial inoculation was characterized by the activation of defense genes (Fig. 2). Eight plant PR proteins were activated. Six of them belonged to the proteinase inhibitor class (PIs, PR-6 family), and the other two were plant defensins (PR-12 family). Likewise, Xcc infection caused a vast array of molecular reactions, including the accumulation of plant defensive secondary metabolites. In this case, different genes implicated in GSL production/degradation were upregulated. Some of these genes were involved in the biosynthesis of the core GSL structure, such as CYP79B3 and CYP79B2, which encode the enzymes responsible for the step from l-tryptophan to indole-3-acetaldehyde oxime during the synthesis of glucobrassicin (GBS) and its derivatives. Furthermore, one gene encoding a myrosinase-associated protein was overexpressed, suggesting that the GSL hydrolysis system was activated.

### Transcriptional reprogramming at 12 dpi

**Fig. 3:**
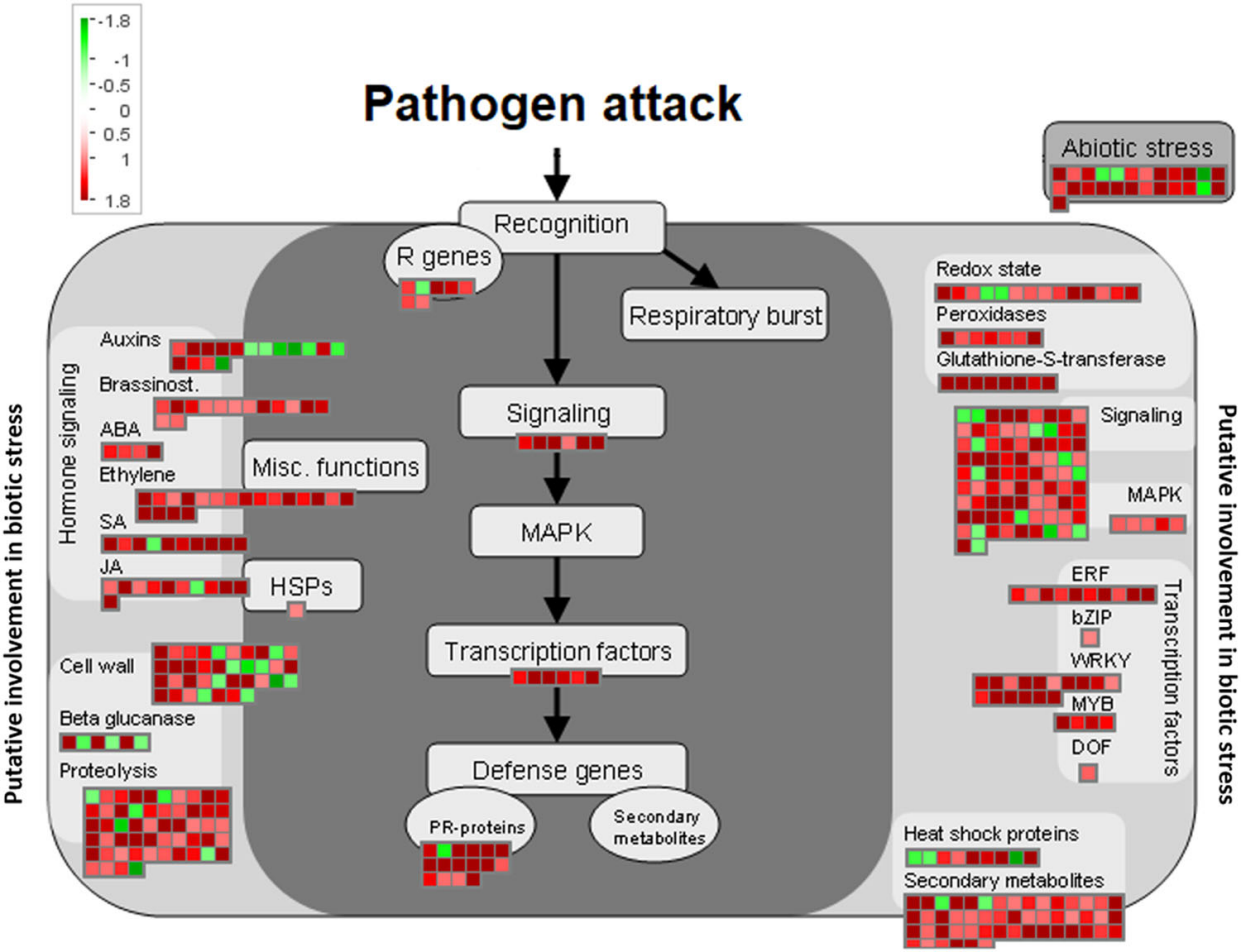
Distribution of the differentially expressed transcripts at 12 dpi that are involved in biotic stress processes by using Mapman software. Green square: downregulated genes; red square: upregulated genes

Twelve days after Xcc colonization, plants exhibited cell death at the infection site followed by the spread of chlorosis and secondary necrosis to the surrounding uninfected tissue. In addition to the processes triggered at 3 dpi, a broad range of other complex mechanisms were activated at 12 dpi (Fig. 3). It is commonly accepted that to trigger the basal defense mechanisms, plants need to detect the presence of the pathogen or the damage produced due to pathogen activity. Our results showed that *B. oleracea* activated the transcription of several kinds of receptors at 12 dpi. Among the different receptor-like genes overexpressed, ~40 belonged to the receptor-like kinase (RLK) gene family, one of the largest gene families encoded by plant genomes^14^. In addition to RLK induction, other related perception mechanisms were activated at this infection point. The intracellular nucleotide-binding/leucine-rich-repeat (TIR-NB-LRR) proteins are involved in the recognition of pathogen effectors or their activity, and therefore, subsequent ETI activation^15^. Surprisingly, whereas the *NB-LRR at1g72870-homolog* gene was significantly overexpressed, *NB-LRR at5g18350-homolog* was repressed. These results indicate a simultaneous induction of factors related to the two branches of plant immunity.

Our results showed that the transcription of different genes encoding redox state-related enzymes was modified by Xcc infection, such as several thioredoxin transferases and peroxidases (class III peroxidases or ascorbate peroxidases). Both ascorbate peroxidase genes detected were repressed. In addition, 17 genes encoding calcium-signaling proteins were overexpressed. Most of these proteins are calcium sensors, which are essential factors for Ca^+2^ transport^11^. Members of the two calcium sensor types (sensor relays and sensor responders) were upregulated during the infection progression.

### Analysis of the calcium-signaling response to Xcc infection

**Fig. 4:**
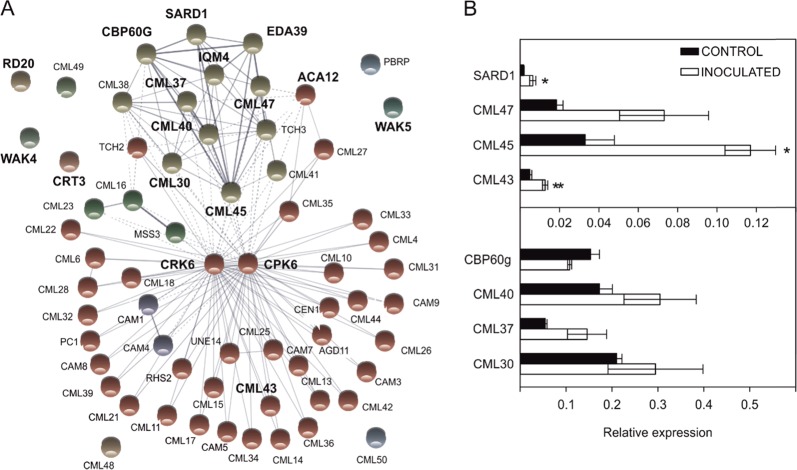
Calcium-signaling transcripts in response to *Xanthomonas campestris* pv. *campestris* infection. **a** Protein association network built with all induced calcium-signaling proteins and the known calcium sensors in Arabidopsis. Bolded names are the differentially expressed genes detected at 12 dpi. Line thickness indicates the strength of the data support, which is based on experimental results and coexpression analysis. **b** Relative expression patterns of different calcium-signaling-related genes from the “Early Big” inbred line at 12 dpi. Data are the average of three biological replicates ± SE. *P-value < 0.05 (Student’s *t* test, Con*t*rol vs. Inoculated)

To further investigate calcium signaling in the *B. oleracea*-Xcc compatible interaction, we evaluated the relationship between the calcium-signaling proteins induced at 12 dpi by using the webtool STRING (v10.5) and setting other known calcium sensors in Arabidopsis as queries (Fig. 4a). The protein association network obtained showed a main functional module that formed a tightly connected cluster. Most of the calcium-signaling proteins differentially expressed between conditions (11 of 17 genes) were responsible for the formation of this major functional module, suggesting that they are part of the same highly connected signaling pathway. To confirm the MACE results, quantitative reverse-transcription-PCR (RT-qPCR) was employed to analyze the expression patterns of 10 of these 17 genes. This selection encompassed members of all known calcium sensor families and other proteins directly related to them. We obtained consistent results for eight of them, and only three genes presented significant differences between conditions, all of which showed a clear tendency (Fig. 4b).

In addition, the MACE results showed that the expression of four genes classified as CaM-binding proteins changed at 12 dpi. Among them, *CAM-BINDING PROTEIN 60* *g* (*CBP60g*) and *SYSTEMIC ACQUIRED RESISTANCE DEFICIENT 1* (*SARD1*) are highlighted since they are two master transcription factors (TFs) of plant immunity. According to the literature, these genes have a partially redundant role^16^.

### Expression of SARD1 and CBP60g in compatible and incompatible Xcc–*B. oleracea* interactions

**Fig. 5:**
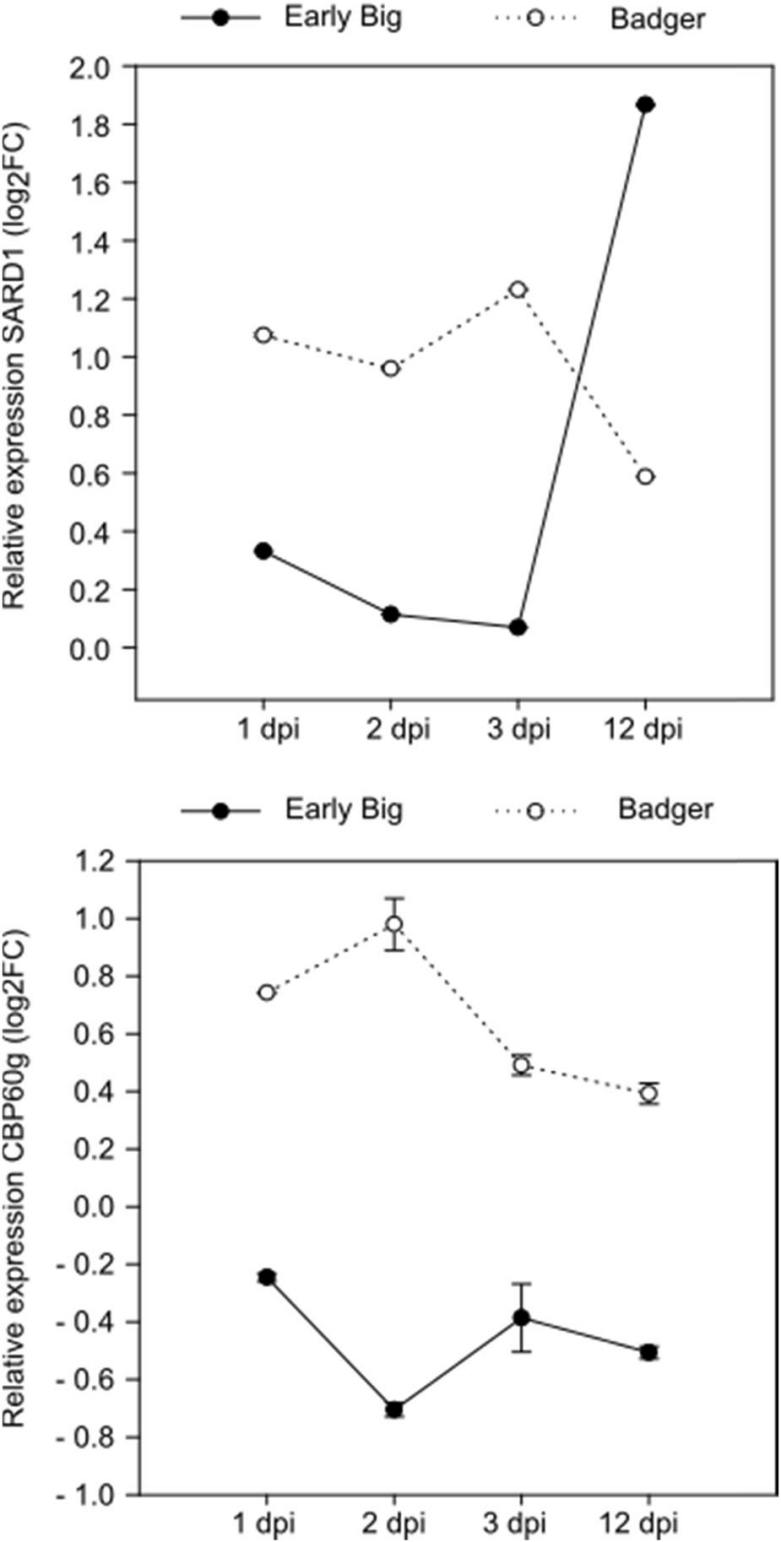
Relative expression patterns of SARD1 and CBP60g in two different *B. oleracea* lines (“Early Big” and Badger Inbred-16, susceptible and resistant to *Xanthomonas campestris* pv. *campestris*, respectively) at four different dpi. Data are the average of three biological replicates. FC was calculated as inoculated/control. Propagation of error was used to calculate uncertainty

To further study the roles of *CBP60g* and *SARD1* in resistance to Xcc pathogenesis, we investigated their expression patterns in two different *B. oleracea* genotypes, one compatible (“Early Big”, used for the MACE analyses) and one incompatible (Badger Inbred-16), at 1, 2, 3, and 12 dpi (Fig. 5). The *SARD1* qPCR results showed a clear difference between the two genotypes. The susceptible line showed a unique overexpression peak at 12 dpi, while the expression level in the resistant line was higher and constant, showing even a slight decrease at 12 dpi. The comparison of *CBP60g* expression between lines offered a striking image. The expression patterns formed a specular image in both genotypes. The expression of *CBP60g* was low during all days postinoculation (dpi) analyzed in the susceptible line, while Badger Inbred-16 maintained a constant and higher level of *CBP60g* expression. Therefore, it appears that the susceptible line is not able to activate *CBP60g* expression or not at a sufficient amount, and therefore, this calcium-signaling branch cascade is interrupted.

### Role of SARD1 and CBP60g in Xcc resistance

**Fig. 6:**
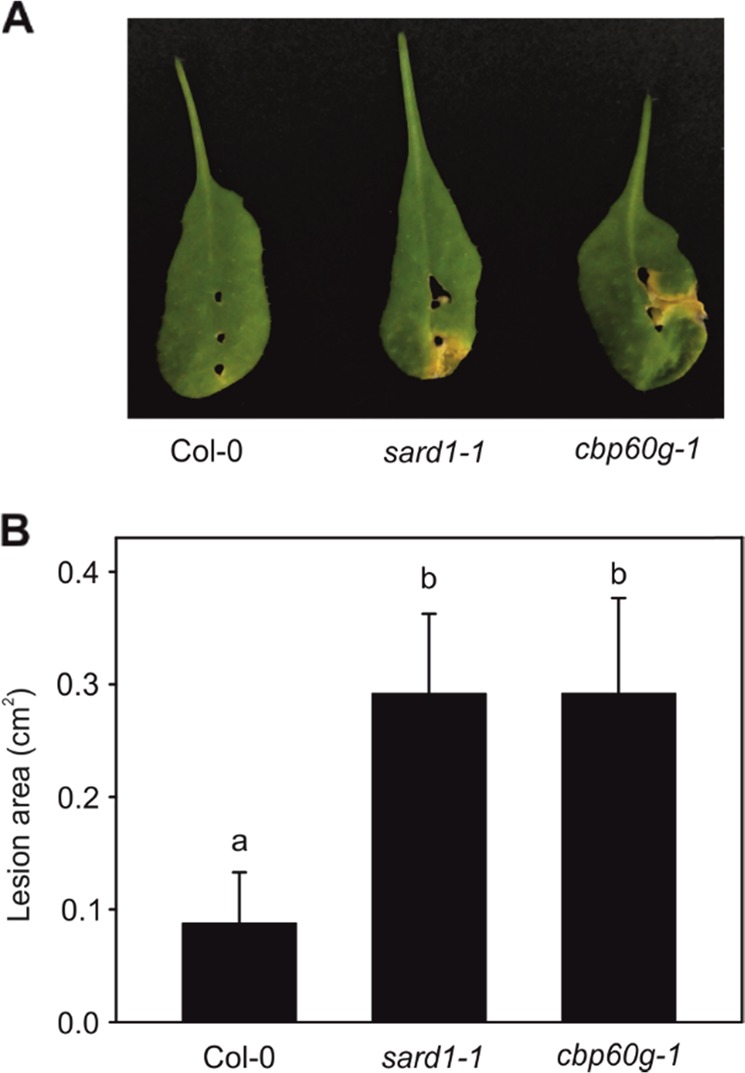
Mutants on the *SARD1* and *CBP60G* genes in *Arabidopsis thaliana* show higher susceptibility to *Xanthomonas campestris* infection than wt. **a** Symptoms on the leaves of wt (Col-0) and *sard1-1* and *cbp60g* mutants. **b** Quantification of the lesion area in the three genotypes. Data are the means of at least seven biological replicates ± SE. Means with different letters are significantly different (Fisher’s protected LSD, *P-*value < 0.05)

To determine whether the role of *SARD1* and *CBP60g* is essential to promote plant resistance to Xcc, we evaluated the response of two Arabidopsis mutants, *sard1-1* and *cbp60g-1*, to infection. We carried out the inoculation with the Xcc race 3 strain HRI5212 since the Arabidopsis plants did not show any symptoms to the infection with Xcc race 1 (even at the susceptible ecotype Sf2)^17^ (data not shown). Typical Xcc necrotic symptoms were clearly visible on the mutant leaves 7 days post infection, whereas Col-0 (wt) barely showed necrotic lesions (Fig. 6a). The area of the infected region was measured using ImageJ software. The statistical analysis of these data confirmed that the necrotic lesions were significantly larger in the two mutants than in wt (Fig. 6b). This result clearly supports our hypothesis that the *SARD1* and *CBP60g* genes play essential roles in plant resistance to Xcc.

## Discussion

To investigate the dynamics of the transcriptional response of *B. oleracea* plants challenged with Xcc, we used MACE technology, a high-resolution and cost efficient RNA-seq variant. Although extensive transcriptomic plant reorganization has been demonstrated to take place a few hours after pathogen perception, we observed a higher response at 12 dpi than at 3 dpi. This could be explained since the plant genotype used in this analysis is a compatible host for Xcc infection. Although susceptible hosts possess basal defense mechanisms, this response could be delayed in comparison with the response of an incompatible host. Generally, this delay is produced by the host’s inability to immediately recognize the invader and therefore produce the proper response^18^. Thus, 3 dpi may not be enough to observe a complete elicited response in our host-pathogen system. This is supported by the fact that we did not observe the activation of some common genes typically involved in immune system activation (i.e., factors responsible for direct or indirect pathogen perception or members implicated in cell-to-cell signaling pathways) during the first days after bacterial infection.

In our conditions, the basal defensive response to the attack of Xcc was characterized by the activation of PR proteins belonging to two major protein families (PR-6 and PR-12). The PR-6 proteins are involved in the reduction of the ability of the pathogen to complete its replication cycle, whereas the role of PR-12 remains unclear^19^. The accumulation of PR proteins is a typical response in plants exposed to both biotic and abiotic stress^20^. Likewise, Xcc infection causes the accumulation of transcripts of plant defensive secondary metabolites. Among these transcripts, those involved in GSL core structure biosynthesis were notable. Specifically, two of these transcripts encode important enzymes involved in the synthesis of GBS. Several authors have studied the role of GSLs or their autolytic breakdown products (isothiocyanates, ITCs) in the defense against Xcc^21,22^. These studies have shown that different ITCs and GSLs, such as gluconapin, sinigrin and sinalbin, had an antibiotic effect against Xcc; however, the GBS biocide effect against Xcc was weaker than that observed in other GSLs. Moreover, the quantification of the content of GSLs in our samples did not show significant differences between the control vs. inoculated plants.

This basal immune response seems to be mediated by phytohormones. It is well known that proper regulation of the immune response is essential to maintain an appropriate energy balance to reduce the inherent fitness cost of being well defended, which is precisely the main role of the phytohormones^23^. Generally, the SA pathway is related to plant defenses against biotrophic pathogens, whereas ethylene (ET) and JA are required to fight against necrotrophic pathogens. However, some authors debate whether pathogens are not often readily classifiable as purely biotrophic or necrotrophic, and the antagonistic or synergistic interactions between SA and JA/ET pathways depend on the specific pathogen and its lifestyle^24,25^. Our results showed an upregulation of genes related to the biosynthesis of these phytohormones, which reinforces the hypothesis that Xcc is not a total biotrophic or necrotrophic bacterium. Markedly, a higher number of transcripts involved in phytohormone metabolism and perception were activated at 12 dpi than at 3 dpi. The role of phytohormones in the long-term immune response has been neglected in the literature.

The plant immune system is composed of different phases in which several factors collaborate together with the aim of curbing pathogen progression. This system is conventionally divided into two interconnected branches: an earlier step called the PTI and the ETI, a later and amplified response that results in a hypersensitive cell death response (HR) at the infection site^2^. Despite this conventional zigzag model presenting ETI and PTI as two well-differentiated branches of plant immunity, recent evidence indicates that they have more in common than previously thought. It appears that depending on the specific plant-pathogen interaction, pattern recognition receptors (PRRs) and effector receptors can swap roles, and most defense mechanisms triggered by plants, such as oxidative burst, hormonal changes, HR or transcriptional reprogramming, can be produced by both responses^8^. Approximately 40 of the pathogen receptors that we identified at 12 dpi belong to the RLK gene family. Most of the members of the RLKs act as PRRs involved in the recognition of PAMPs in the plasma membrane, which is essential to trigger PTI^26^. We found PRR genes with different kinds of ectodomains, the epidermal growth factor-like domain, lectin and lysine motifs or LRRs, each involved in the recognition of specific PAMPs during pathogen invasion. Thus, although Xcc infection was well established and plants presented high levels of damage, plants were still triggering the mechanisms related to pathogen perception, which are processes generally associated with the early stages of basal immunity.

The perception of pathogen invasion produces the activation of multifaceted intracellular signaling pathways that initiate defense responses. The role of hydrogen peroxide (H_2_O_2_) as a second messenger is well known; if the organism is subjected to increasing levels of H_2_O_2_, as observed during pathogen attack, this signal must be propagated to trigger the appropriate responses^27^. Generally, this oxidative burst is accompanied by changes in the intracellular environment, such as fluxes in Ca^+2 28^, which acts as a pleiotropic second messenger to trigger numerous physiological processes. However, the mechanism of this essential signaling pathway remains unclear due to the cellular location and nature of these calcium signals that differ across both host species and pathogenesis, which probably reflects mechanistically distinct processes^29^.

CMLs, which are solely present in the plant kingdom, display a strong affinity for calcium ions. The interaction between a Ca^+2^ ion and one CML induces conformational changes that trigger its association with downstream target proteins^11^. CML targets include protein kinases, metabolic enzymes, transporters and transcription factors. Although the role of most of these CMLs remains unclear, some CMLs are stress responsive. For example, *CML37* showed dual roles in biotic and abiotic responses, acting as a positive regulator of defense against herbivores and a negative regulator during drought stress tolerance^11^. *CML42* is both a negative regulator of insect herbivory-induced defense and drought-induced ABA levels and a positive regulator of UV stress. Our in silico study focused on members of the calcium-signaling pathway suggests, for the first time, the possible implication of *CML30*, *CML37*, *CML40*, *CML43*, *CML45*, and *CML47* in the response against pathogenesis.

Since these proteins appear to act as Ca^+2^ flow transmitters, most of the studies are focused on the identification and characterization of their downstream targets^30^. Among these genes, we identified two master transcription factors of plant immunity, *CBP60g* and *SARD1*. A study performed by Wang et al.^31^ using Arabidopsis *cbp60g* mutants showed that this protein contributes to PAMP-triggered SA accumulation, which enhances resistance against *P. syringae*. In addition to SA-dependent defense pathway activation, both *CBP60g* and *SARD1* activate SA-independent defense mechanisms through the regulation of WRKY70 expression^32^. In fact, in this work, we reported the overexpression of WYRK70 at 12 dpi, which is further indication of the action of these transcription factors. Interestingly, in light of our results, the expression patterns of *CBP60g* and *SARD1* are opposing. Despite these genes belonging to the same protein family, it has been shown that their regulation is different. *CBP60g* necessarily requires CML binding, and *SARD1* provides a similar role in a Ca^+2^-independent manner^33^. Therefore, strictly speaking, *SARD1* should not be considered a CaM-binding protein and indicates that the functions *CBP60g* and *SARD1* are carried out in parallel^32^. This fact could explain their different expression patterns during Xcc infection.

The comparison of the expression of these genes between a compatible and an incompatible genotype suggests an essential role in the resistance against Xcc. The fact that Arabidopsis knockout mutants of these genes are more susceptible to Xcc infection than wt plants confirms this role. Apparently, both *SARD1* and *CBP60g* are important during defense against Xcc. A time-course analysis of the expression of these genes suggests that *SARD1* is induced too late in the compatible genotype when the pathogen is already spread through the whole plant. In addition, the incompatible genotype may not need a sharp increase in *SARD1* expression, since this genotype presents a higher basal expression level.

In summary, the MACE results provide a complete view of the variation in the expression of genes potentially involved in the *B. oleracea*–Xcc interaction. In general, we identified more induced than repressed transcripts at both dpi analyzed, albeit the response was greater at 12 dpi. In addition, in contrast to expectations, changes in the expression of genes related to early infection stages, such as PAMP perception or ROS burst and Ca^+2^ flux signaling, were only detected at 12 dpi. Our results suggest that several CMLs could have an important role during Xcc pathogenesis and that the genes *CBP60g* and *SARD1* act as downstream factors of Ca^+2^ signaling.

## Material and methods

### Plant material

The doubled haploid broccoli line “Early Big” (*B. oleracea* var. *italica*) was used for transcriptomic analysis. The Badger Inbred-16 line was subsequently used as a tester of resistance. This line presents a partial black rot-resistance, which is controlled by a quantitative trait locus (QTL). Plants were sown in plastic pots containing *sphagnum* peat (GRAMOFLOR GmbH & Co, Vechta, Germany) in a greenhouse with a minimum temperature of 20 °C during the day and 15 °C during the night, venting at 25 °C and 60% humidity.

### Inoculation of *B.oleracea*

Xcc race 1 strain HRI3811 was provided by Warwick HRI (Wellesbourne, UK). Bacterial cultures were grown in screening media 523 (Sigma-Aldrich, St. Louis, MO, USA) at 30 °C in a rotary incubator at 100 rpm for 48 h. An aliquot was diluted in sterile water to reach a final absorbance of 0.5, which corresponds to a concentration of 5 × 10^8^ cfu/ml. The turbidity of the suspension was measured with a microplate spectrophotometer (Spectra MR; Dynex Technologies, Chantilly, VA, USA) at a wavelength of 600 nm. Plants at the six-leaf stage were inoculated at the third leaf following the method described by Lema et al.^34^. Briefly, a sterilized “florists’ frog” (i.e., multiple needles mounted in circles) was used for inoculation by pressing through the leaf onto a sponge submerged in the inoculum at the edge of the distal side of the leaf. Control plants were mock inoculated following the same procedure. After inoculation, greenhouse conditions were changed to maintain a minimum temperature of 18 °C and an 80% relative humidity. The whole inoculated leaf was from three independent biological replicates of the control, and inoculated plants were collected at 3 and 12 dpi in liquid nitrogen and conserved at −80 °C until processed.

### Inoculation of *Arabidopsis thaliana*

Arabidopsis plants were grown on sterilized peat in a growth chamber under fluorescent light (228 µmol m^−2^ s^−1^) in short-day conditions (8 h light/16 h darkness) and watered as needed. A constant day/night temperature was set at 20 °C. Xcc race 3 strain HRI5212 was provided by Warwick HRI. Bacterial cultures were prepared as described above. Fully expanded leaves of 6-week-old plants were inoculated as described in Meyer et al.^17^. Briefly, inoculation was performed using a sterilized inoculation needle dipped on bacterial culture. Three inoculation points were established in the midrib of each leaf. After inoculation, plants were covered with a plastic bag to maintain nearly 100% relative humidity. At 7 dpi, inoculated leaves were collected and digitalized with a scanner at 300 dpi resolution. The lesion area of each leaf was calculated using ImageJ software^35^.

### RNA isolation, library preparation, and sequencing

Individual sample tissues were ground in liquid nitrogen, and total RNA from three biological replicates of each treatment was extracted using the Spectrum^TM^ Plant Total RNA kit (Sigma-Aldrich, MO, USA). To remove any traces of genomic DNA, the RNA was treated with DNase according to the manufacturer’s instructions. Massive analysis of 3′-cDNA ends (MACE) was performed for each sample (GenXPro GmbH, Frankfurt am Main, Germany) as described by Zawada et al.^36^. Briefly, poly-adenylated mRNA was isolated from 1 μg of total RNA, and cDNA was produced by first- and second-strand synthesis using the SuperScript^®^ III First-Strand Synthesis System (Invitrogen, Waltham, MA, USA) with modified barcoded poly-T adapters that are biotinylated at the 5′ end. After cDNA random-fragmentation, the 3′-ends were captured by streptavidin beads, and 5′ ends of ≈ 67 bp long fragments from the 12 barcode samples were sequenced (single-read) using an Illumina HiSeq 2000 version 4 chemistry (Illumina, Inc., San Diego, CA, USA), with 1 × 125 bps (6 bps were used for a barcode). To eliminate PCR-based copies from the generated dataset, GenXPro’s “TrueQuant” method was applied. This method identifies and eliminates copies with an identical barcode-sequence combination^37^. The average raw count of each gene within a library was normalized by dividing by the geometric mean of all counts in all samples, and the median of the quotients was calculated per library. Each raw count was then divided by the library-specific median value. Statistical analysis was performed using the DEseq R package according to Anders and Huber recommendations^38^.

### Transcript annotation and functional analysis

Putative functions were assigned to the resulting transcripts by mapping in silico their translated protein sequences to the UniProtKB/Swiss-Prot, UniProtKB and Ref-Seq protein databases using the BLASTX algorithm available at NCBI in hierarchical manner, using an *e*-value of 10^−5^ as a threshold for considering them as homologs. Transcripts without homology to these sequences were subsequently annotated to the nonredundant nucleotide NCBI database (nr) by BLASTN using an *e*-value of 0.001 as a threshold.

Transcripts with a false discovery rate <0.05 and −1 < log_2_ fold change (FC) > 1 were considered to be differentially expressed between the control and inoculated samples. The functional classification of the differentially expressed genes was performed with MapMan 3.6.0RC1 software by using *A. thaliana* homolog genes as input. This tool allows the data to be organized according to Gene Ontologies (GO) and displayed by the user in the context of preexisting biological knowledge^39^. Furthermore, the webtool STRING (v. 10.5) was used to study the interconnections between the selected genes. This resource, in addition to the well-supported protein–protein interactions experimentally observed, includes indirect and predicted interactions on top^40^.

The transcriptomic data can be found in the Gene Expression Omnibus repository with the accession number GSE107720.

### Quantitative reverse-transcription-PCR (RT-qPCR) validation

Plant from the inbred lines “Early Big” and Badger Inbred-16 were grown in the greenhouse as described above. Leaves from three biological replicates of each line were gathered at different infection points (1, 2, 3, and 12 dpi). The procedure for RNA extraction was the same as that followed in the MACE analysis. Three technical replicates were performed for each biological replicate. All primer pairs used are listed in Table S1. One microgram of total RNA was reverse transcribed using the GoScript™ reverse-transcription system and oligo (dT20) (Promega, Madison, WI, USA). RT-qPCR was performed in a 20 μl reaction with the Fast Start Universe SYBR Green Master (ROX) mix (Roche Molecular Systems Inc, Pleasanton, CA, USA), following the manufacturer’s instructions. The glyceraldehyde-3-phosphate-dehydrogenase transcript was used as housekeeping gene^41^. RT-qPCRs were carried out on a 7500 Real-Time PCR System (Applied Biosystem, Forster City, CA, USA), and primer efficiency was calculated using the LingRegPCR software^42^. Efficiencies were used to calculate relative gene expression using the ΔΔCt method^43^. Statistical significance was calculated using a two-tailed Student’s *t* test to compare the relative gene expression in the control vs. inoculated plants.

## Supplementary information

Supplementatary Information
