# Supplementary material for: Calcium-signaling proteins mediate the plant transcriptomic response during a well-established Xanthomonas campestris pv. campestris infection
Source: Hortic Res. 2019 Sep 11;6:103. doi: 10.1038/s41438-019-0186-7 (PMC6804691; doi:10.1038/s41438-019-0186-7)
Supplement: Supplementary file 1 — Supplementatary Information [file 41438_2019_186_MOESM1_ESM.pdf]

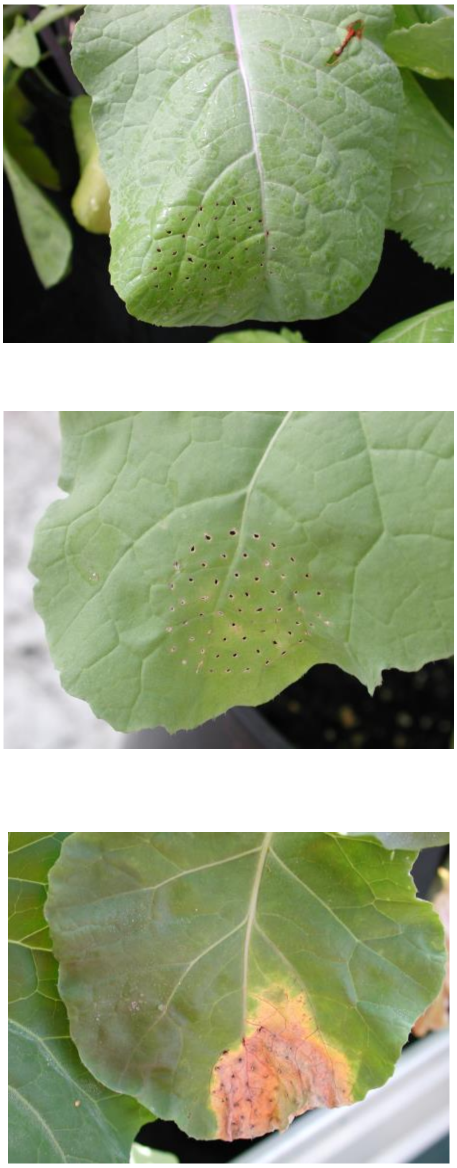

Figure S1. Symptoms of *Xanthomonas campestris* infection in *Brassica oleracea* leaves. dpi: days post-infection.

Table S1. Primers used for RT-qPCR

Gene	Forward	Reverse
Systemic acquired resistance (SAR) Deficient 1 (SARD1)	AGCCCTAGAGCCTGTTCTGA	ACTCTCCCGAGCTTCAACAC
Calmodulin-binding protein 60g (CBP60g)	GACCAAAGTGTTCATCGGCG	CTACCGTCCCTAAAGCGGTC
Calmodulin-like 30 (CLM30)	GGGAGATGGGTTTCTTCTGCT	CACAGTCTGGTCTTGGTTCCT
Calmodulin-like 37 (CLM37)	CGGATGTATGTGACGGAGGG	TGCATCAACCGTACACGACT
Calmodulin-like 40 (CLM40)	ACGTGAAGAGTATCAACGGGT	GTACGACCTTTTCCCCGGAC
Calmodulin-like 43 (CLM43)	GACGAGTCCTTTTTCGCAGG	CAACTCCACGGCGGAGATAA
Calmodulin-like 45 (CLM45)	GTCCAGAGCTCCGTCAAACA	CGTTTCTGAAGTCCCTCGCT
Calmodulin-like 47 (CLM47)	TCGTCCCAGCTCAGGTAGAA	CCTTACCCATCACCTCGCTC
